# Supplementary material for: Human embryonic stem cells secrete macrophage migration inhibitory factor: A novel finding
Source: PLoS One. 2023 Aug 24;18(8):e0288281. doi: 10.1371/journal.pone.0288281 (PMC10449177; doi:10.1371/journal.pone.0288281)

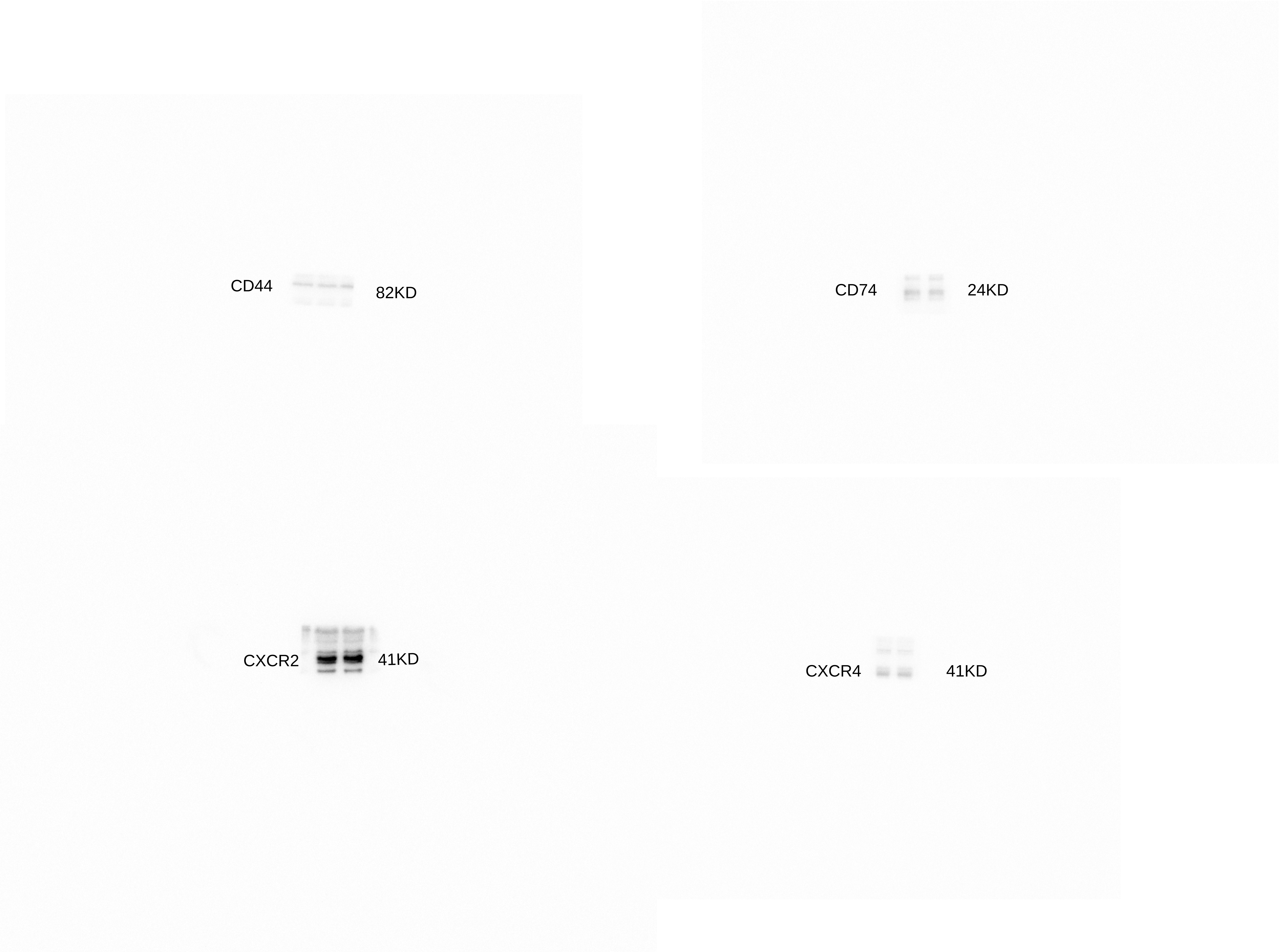
CD44
82KD
CD74
24KD
CXCR2
41KD
CXCR4
41KD

CXCR7 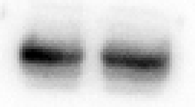 41KD

MIF 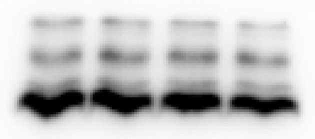 13KD

Tubulin 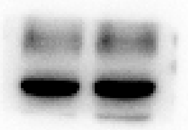 50KD

figure 4 c

CXCR7 IgG Input

CXCR7 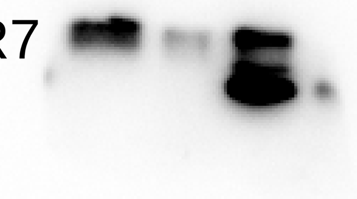 41KD

Figure4 C

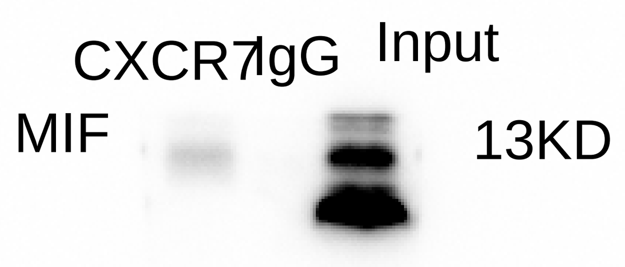

Figure4 B

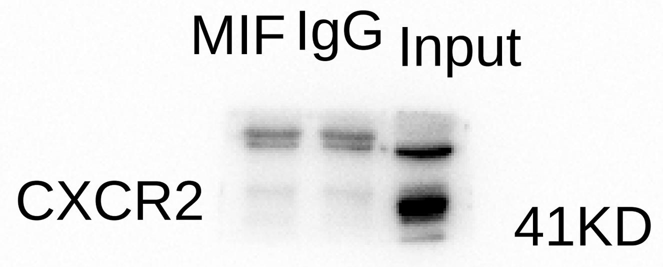

Figure4 A

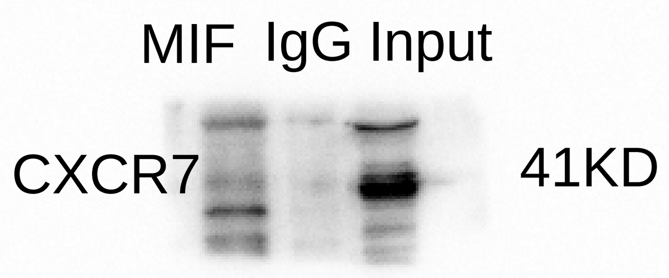

Figure4 A

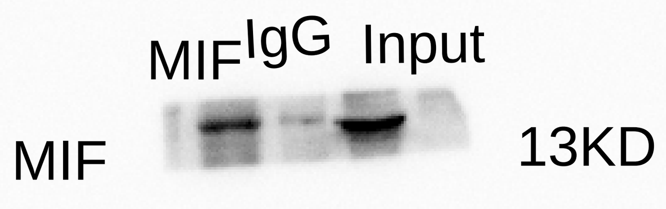

Supplement: S1 Raw images — (PDF) [file pone.0288281.s001.pdf]
