# Supplementary material for: Human embryonic stem cells secrete macrophage migration inhibitory factor: A novel finding
Source: PLoS One. 2023 Aug 24;18(8):e0288281. doi: 10.1371/journal.pone.0288281 (PMC10449177; doi:10.1371/journal.pone.0288281)
Supplement: Attachment — Submitted filename: Response to Reviewers.docx [file pone.0288281.s002.docx]

**Response to Academic Editors**

1. This revision has been revised according to the PLOS ONE style template.
2. English abstracts have been provided.
3. The funding information has been changed in Edit Manager and the grant number has been verified.
4. Author contributions:

Conceptualization: zhenqiang zhao, Tan Wang

Data curation: Yanzhao Wei, Xiaohan Zheng, Yuanji Zhong, Xufang Wei

Formal analysis: Yanzhao Wei, Xiaohan Zheng, Ting Huang

Funding acquisition: zhenqiang zhao

Investigation: Yanzhao Wei, Xiaohan Zheng, Xufang Wei

Methodology: Yanzhao Wei, Xiaohan Zheng, Yuanji Zhong, Qibing Liu

Project administration: zhenqiang zhao, Tan Wang, Yanzhao Wei, Xiaohan Zheng

Resources: zhenqiang zhao, Tan Wang, Qibing Liu

Software: Yanzhao Wei, Xiaohan Zheng, Ting Huang, Yuanji Zhong

Supervision: zhenqiang zhao, Yanzhao Wei, Xiaohan Zheng

Validation: Ting Huang, Yuanji Zhong, Shengtong Sun

Visualization: Ting Huang, Yuanji Zhong, Shengtong Sun, Xufang Wei

Writing-original draft: zhenqiang zhao, Yanzhao Wei

Writing-review editing: zhenqiang zhao, Tan Wang, Xiaohan Zheng

5. The grant-related text in the manuscript has been removed. Our funding statement is amended as follows：

This work was supported by a grant from National Natural Science Foundation of China (grant number 81860238); Hainan Provincial Natural Science Foundation of China (grant number 821RC694); Major Research and Development Project of Hainan Province of China (grant number ZDYF2018233); Scientific research projects in Colleges and Universities of Hainan Province of China (grant number HYYS2021A19); Project of Hainan Province Clinical Medical Center. The funders had no role in study design, data collection and analysis, decision to publish, or preparation of the manuscript. Modified text is marked in blue.

6. “The authors have declared that no competing interests exist.” has stated in the cover letter. Modified text is marked in blue.
7. Data availability is modified to “All relevant data are within the paper and its Supporting information files.” Modified text is marked in blue.
8. blot/gel image data has been added to the supporting information
9. ORCID iD of the corresponding author has been added in the Editorial Manager.
10. The title of the supporting information has been added at the end of the manuscript.

**Responses to reviewers**

1. This revision adds a discussion of DDT to the discussion section. Modified text is marked in blue.
2. This revision adds the relationship of MIF to autoimmune diseases to the discussion section. Modified text is marked in blue.
3. The role of MIF in tumorigenesis has been removed due to the deletion of the text mentioned in point 5.
4. Due to the role of MIF in embryonic stem cells, MIF suppression during pregnancy should be evaluated as well. Modified text is marked in blue.
5. The text of this paper has been appropriately abridged to make it more concise.
